# Mesenchymal stem cell‐derived exosomes ameliorate erection by reducing oxidative stress damage of corpus cavernosum in a rat model of artery injury

**DOI:** 10.1111/jcmm.14615

**Published:** 2019-09-11

**Authors:** Yangzhou Liu, Shankun Zhao, Lianmin Luo, Jiamin Wang, Zhiguo Zhu, Qian Xiang, Yihan Deng, Zhigang Zhao

**Affiliations:** ^1^ Department of Urology & Andrology, Minimally Invasive Surgery Center, Guangdong Provincial Key Laboratory of Urology The First Affiliated Hospital of Guangzhou Medical University Guangzhou China; ^2^ Department of Urology Zhejiang Taizhou Central Hospital (Taizhou University Hospital) Taizhou China

**Keywords:** artery injury, cavernous sinus endothelial cells, erectile dysfunction, exosomes, mesenchymal stem cells, oxidative stress damage

## Abstract

Erectile dysfunction (ED) is a common ageing male's disease, and vascular ED accounts for the largest proportion of all types of ED. One of the mechanisms of vascular ED in the clinic is arterial insufficiency, which mainly caused by atherosclerosis, trauma and surgical. Moreover, oxidative stress damage after tissue ischemia usually aggravated the progress of ED. As a new way of acellular therapy, mesenchymal stem cell‐derived exosomes (MSC‐Exos) have great potential in ED treatment. In the current study, we have explored the mechanism of MSC‐Exos therapy in a rat model of internal iliac artery injury‐induced ED. Compared with intracavernous (IC) injection of phosphate‐buffered saline after artery injury, of note, we observed that both mesenchymal stem cells (MSCs) and MSC‐Exos through IC injection could improve the erectile function to varying degrees. More specifically, IC injection MSC‐Exos could promote cavernous sinus endothelial formation, reduce the organization oxidative stress damage, and improve the nitric oxide synthase and smooth muscle content in the corpus cavernosum. With similar potency compared with the stem cell therapy and other unique advantages, IC injection of MSC‐ Exos could be an effective treatment to ameliorate erectile function in a rat model of arterial injury.

## 1 INTRODUCTION

As one of the male sexual disorders, erectile dysfunction (ED) is considered a common disease in the ageing male. According to an early research, the prevalence rate of ED in men aged 40‐70 was 52%,^1^ and the number of ED worldwide is predicted to increase to 322 million in 2025.^2^ Penile erection is a comprehensive result of nerve mobilization, arterial blood supply and cavernous blood storage. Vascular ED accounts for the largest proportion of all types of ED, accounting for about 50% of all types of ED in people over 50 years old.^3^ Common mechanism of vascular ED in the clinic is arterial insufficiency, which mainly caused by atherosclerosis, trauma and surgical. Many studies have proved that in arterial ED, as the corpus cavernosum tissue continues to be stimulated by the ischemic and hypoxic environment, the release of reactive oxygen species in the penis increases. When the harmful compounds produce more than the anti‐oxidative defence ability of the tissue, free radical accumulation may cause cell degeneration, cavernosum vascular endothelium and nerve damage, thereby further increasing the progress of ED.^4^, ^5^, ^6^ Previous studies have proved that antioxidant therapy is of great significance for arterial ED.^7^

Phosphodiesterase type 5 inhibitors (PDE5is) are the first‐line treatment for ED. However, the efficiency of PDE5is must base on the integrity of corpus cavernosum vascular endothelial function and bioavailability of the nitric oxide (NO).^8^ Given these reasons, it is difficult to achieve satisfactory results for those patients with more serious refractory ED. On the other hand, PDE5is are expensive and have serious adverse effects.^9^ Thus, it is necessary for exploring other strategies with better efficacy for treating ED, especially for arterial ED.

Bone mesenchymal stem cells (MSCs) are pluripotent cells with strong differentiation ability under different conditions. Stem cells were originally considered as substitutes for defective or damaged cells in the past. More recently, however, various types of stem cells have proven to influence the host environment due to they can secrete various bioactive molecules by the paracrine route.^10^, ^11^ Stem cells could secrete a large number of biological factors, which have nutrition, angiogenetic, antifibrotic and inflammatory modulating properties. Moreover, as a method of stimulating the activity of tissue resident receptor cells, stem cells are also able to secrete nucleic acids, lipids and proteins in the extracellular microvesicles.^12^, ^13^, ^14^

The exosomes mainly refer to particles between 40 and 150 nm in diameter among the many types of extracellular microvesicles.^15^ According to the latest research, exosomes derived from stem cell are efficacious in animal models of ED, which contains acute cavernous nerve injury^16^ and type 2 diabetes.^17^ However, whether transplant MSC‐derived exosomes (MSC‐Exos) can be exploited to recover arterial injury ED, and the mechanisms remain largely undetermined. As tissue will continue to be exposed to risk factors during vascular events, compare with the protective effect of exosomes on anti‐apoptosis after tissue injury,^16^, ^17^ we found that this therapeutic effect might mainly delay the damage of cavernosum by promoting tissue repair. In the current study, we have explored the treatment efficacy of intracavernous (IC) injection of MSCs or MSC‐Exos in a rat model of arterial injury ED and further elucidate the underlying mechanism of MSC‐Exos treatment.

## 2 MATERIALS AND METHODS

### 2.1 Animal models and experimental design

A total of 30 male Sprague Dawley (SD) rats (12 weeks old, 300‐350 g) were obtained from the Laboratory Animal Center of Guangzhou University of Chinese Medicine. The animals were kept on a 12‐hours light/dark cycle at the Center for Experimental Animals at the First Affiliated Hospital of Guangzhou Medical University. All experimental protocols were approved by the Institutional Animal Care and Use Committee at the First Affiliated Hospital of Guangzhou Medical University.

All the rats were randomly divided into five groups of six rats each. The sham group had low abdominal incision but without internal iliac artery ligation. The remaining animals were subjected to bilateral internal iliac arteries ligation to establish arteriogenic ED models.^18^ Four weeks later, the 24 rats were randomly divided into four groups. The PBS group received IC injection of phosphate‐buffered saline (PBS); the MSCs and MSC‐Exos groups received IC injection of MSC or MSC‐Exo separately. At the fourth week after IC injection, animals' erectile function was measured and the penile tissues were harvested for histologic analysis and western blotting.

### 2.2 Bilateral internal iliac arterial ligation surgery

**Figure 1:**
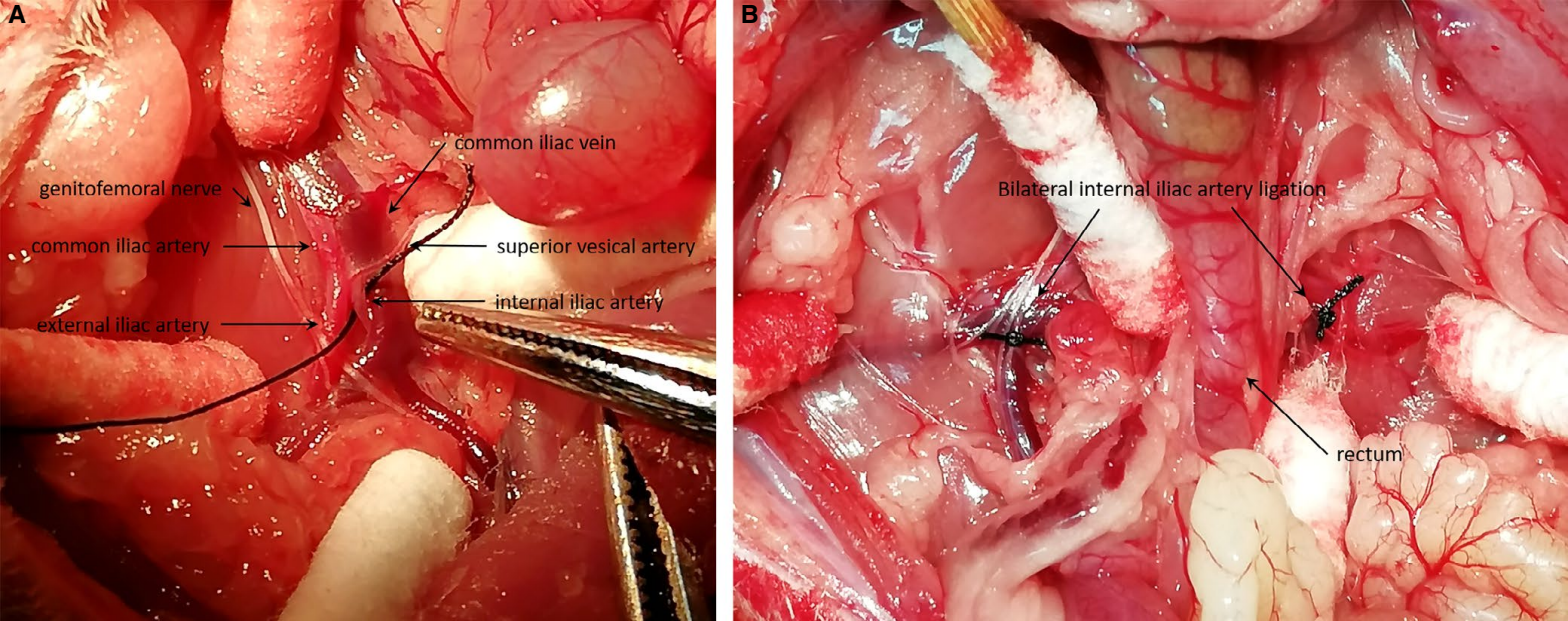
Establishment the rat model of artery injury erectile dysfunction. A, Expose and dissociate the internal iliac artery. B, Bilateral internal iliac arterial ligation

The ligation surgery was performed under anaesthesia with 45 mg/kg pentobarbital sodium, body temperature of each animal remains stable. A 4‐5 cm transverse incision in lower abdomen was made, then under a dissecting microscope, deep below the superior vesical artery surface free internal iliac artery (Figure 1A). After bilateral internal iliac arteries were ligated (Figure 1B), the organ reduction and incision was sutured. Intramuscular injection of 210 mg/kg/d cefazolin sodium pentahydrate to prevent infection in the first 3 days after surgery.

### 2.3 Isolation and identification of MSC‐exos

Sprague Dawley rat bone MSCs line (Cat. No SCSP‐ 402) was purchased from the Cell Bank of Chinese Academy of Sciences. Mesenchymal stem cells were cultured in Mesenchymal Stem Cell Medium (ScienCell Research Laboratories) with 5 mL of penicillin/streptomycin solution (P/S, Cat. No. 0503); 5 mL of mesenchymal stem cell growth supplement (MSCGS, Cat. No. 7552) and 25 mL of foetal bovine serum (FBS, Cat. No. 0025). Once at 80% confluence, MSCs were starved (use PBS to wash cells for three times, replaced with the serum‐free medium) 24 hours and then the conditioned medium was collected. MSC‐Exos were isolated through multistep centrifugation. Firstly, the medium was centrifuged to eliminate dead cells and debris (300 g for 10 minutes, 2000 g for 20 minutes, and 10 000 g for 30 minutes). Then, the supernatant was transferred to an ultrafiltration centrifuge tube (Millipore) and centrifuged at 3000 g for 10 minutes. Subsequently, the condensed supernatant was filtered through a 0.22‐μm filter to eliminate impurities. Finally, the filtrate was recovered and followed by ultracentrifugation at 120 000 g for 70 minutes to precipitate the exosomes (Thermo Scientific).

The pellet was resuspended in PBS. The quantification of exosomes was determined by the protein concentration using the Bicinchoninic Protein Assay (Thermo Scientific, Pierce^TM^ BCA Protein Assay Kit). Exosomes morphology was further detected by a transmission electron microscopy (JEM‐1200EX, JEOL) analysis, and size distribution analysis was done using the Nanoparticle Tracking Analysis (Nanosight NS300). Exosomes' characteristic marker proteins levels of CD9 (Abcam, ab92726, 1:2000) and TSG101 (Abcam, ab125011, 1:5000) were determined using western blot.

### 2.4 IC injection

For IC injections, a dose of 3 × 10^6^ MSCs from a 75 cm^2^ cell culture flask was administered for one animal. The low‐dose group of MSC‐Exos (50 µg protein) was determined by corresponding to the amount produced by 3 × 10^6^ MSCs equivalents from cell culture supernatants; and the high one (100 µg protein) is the double of the low dose. The MSCs or MSC‐Exos are suspended in 100 µL PBS.

During the operation, the rats were anesthetized with pentobarbital sodium (45 mg/kg). The foreskin is rolled up to expose the penis, then 100 µL PBS (PBS group) or suspensions (MSCs and MSC‐Exos group) were injected into the middle section of the cavernosum tissue. The needle was left in place for 1 minute to allow suspensions diffuse in the cavernosum tissue. The base of the penis was clamped with microscopic vascular clamp (15 mm; Mingmou Medical Apparatus Instruments) to block the local blood circulation for holding the MSCs or MSC‐Exos in corpus cavernosum situ; the clamps were removed at 15 minutes after the injection.

### 2.5 Erectile function evaluation

Erectile function was assessed based on changes in intracavernous pressure (ICP) after the cavernous nerves electrostimulation.^19^ Under anaesthesia with intraperitoneal injection pentobarbital sodium (45 mg/kg), ICP was measured with a 25‐G heparin‐filled needle, which inserted into the corpora cavernosum of the penis. The major pelvic ganglia were identified through a lower abdominal midline incision and then the cavernous nerves were exposed, the animal nerve‐stimulating electrode (3‐mm apart, ADINSTRUMENTS, MLA0320) was connected to the cavernous nerve. The stimulus parameters were as follows: 10‐V amplitude, 15‐Hz frequency, 5‐ms pulse width and 60‐seconds duration. The electrical stimulation was done in triplet, with a 5‐minutes interval between the subsequent stimulation to ensure stability maintains for each animal.^20^ In the meantime, the right internal carotid artery was detached and inserted into a 24‐G vein indwelling needle to measure mean arterial pressure (MAP). All blood pressure change data were recorded by the ADInstruments PowerLab 16/35 workstation and analysed by LabChart 8 Software (ADInstruments). The ratio of maximal ICP to MAP was calculated to normalize for variations in systemic blood pressure.

### 2.6 Histologic analysis

After erectile function was evaluated, the penile midshaft at the injection site was harvested. One‐third of the tissues were fixed in 4% paraformaldehyde, then dehydrated and embedded in paraffin, and the rest fresh tissue was frozen in liquid nitrogen for other assays. The tissues were coronally cut to 5‐µm thickness for immunofluorescence (IF), immunohistochemistry (IHC), haematoxylin and eosin (HE) and Masson's trichrome staining.

Paraffin sections were stained following the standard procedures for histologic study. For IF, double fluorescence staining of tissue sections with primary antibodies mouse anti‐CD31 ( Servicebio, GB12063, 1:300) and rabbit anti‐OCT4 (Abcam, ab18976, 1:50), the secondary antibodies used were anti‐Ms CY3 (Servicebio, GB21301, 1:300) and anti‐Rb FITC (Servicebio, GB22303, 1:300), nuclei were stained with 4‐6‐diamidino‐2‐phenylindole (DAPI, Servicebio, G1012). For IHC, the penile sections were incubated with primary antibodies to anti‐Cu‐Zn superoxide dismutase‐1 (SOD‐1, Santa Cruz Biotechnology, sc‐101523, 1:500), anti‐DNA/RNA damage (8‐OHdG, Abcam, ab62623, 4µg/mL), anti‐alpha smooth muscle actin (α‐SMA, Abcam, ab32575, 1:300). The negative control used PBS to substitute for primary antibody. Brown coloration indicated positive expression. The scoring of immunoreactivity was obtained by staining intensities/ area and positive cell percentages.

Hematoxylin and eosin staining was used to measure the perimeter of cavernosum, and Masson's trichrome staining was used to quantify the ratio between smooth muscle (red) and collagen (blue) in corpus cavernosum. Slices were observed via confocal laser scanning microscopy (LEICA, DMi8, Mannheim/Wetzlar) or scanned by PathScope^TM^ 4S (DigiPath), computerized histomorphometric analyses were implemented using Image‐Pro Plus 6.0 (Media Cybernetics).

### 2.7 Western blotting

Western blot (WB) was performed as previously described.^21^ Primary antibodies include anti‐CD31 (Abcam, ab222783, 1:2000), anti‐VEGFA (Abcam, ab1316, 5 µg/mL), anti‐neuronal nitric oxide synthase (nNOS, Abcam, ab76067, 1:1000), anti‐endothelial nitric oxide synthase (eNOS, Abcam, ab199956, 1:1000), anti‐inducible nitric oxide synthase (iNOS, Abcam, ab178945, 1:1000), anti‐SOD (Santa Cruz Biotechnology, sc‐101523, 1:500), anti‐α‐SMA (Abcam, ab32575, 1:2500). Anti‐GAPDH (Abcam, ab8245, 1:5000) and anti‐tubulin (Abcam, ab210797, 1:1000) were used as the internal standard. The image of protein expression was detected by Odyssey CLX Two‐colour infrared laser imaging system (LI‐COR Biosciences). Densitometric analysis of the bands was implemented using ImageJ software (National Institutes of Health).

### 2.8 Statistical analyses

The results were analysed using GraphPad Prism software (version 7.0, GraphPad Software) and expressed as mean ± standard error of the mean. Multiple groups were compared using one‐way analysis of variance followed by the Student‐Newman‐Keuls test for post hoc comparisons. Statistical significance was set at *P* < .05.

## 3 RESULTS

### 3.1 Characterization of MSCs and MSC‐exos

**Figure 2:**
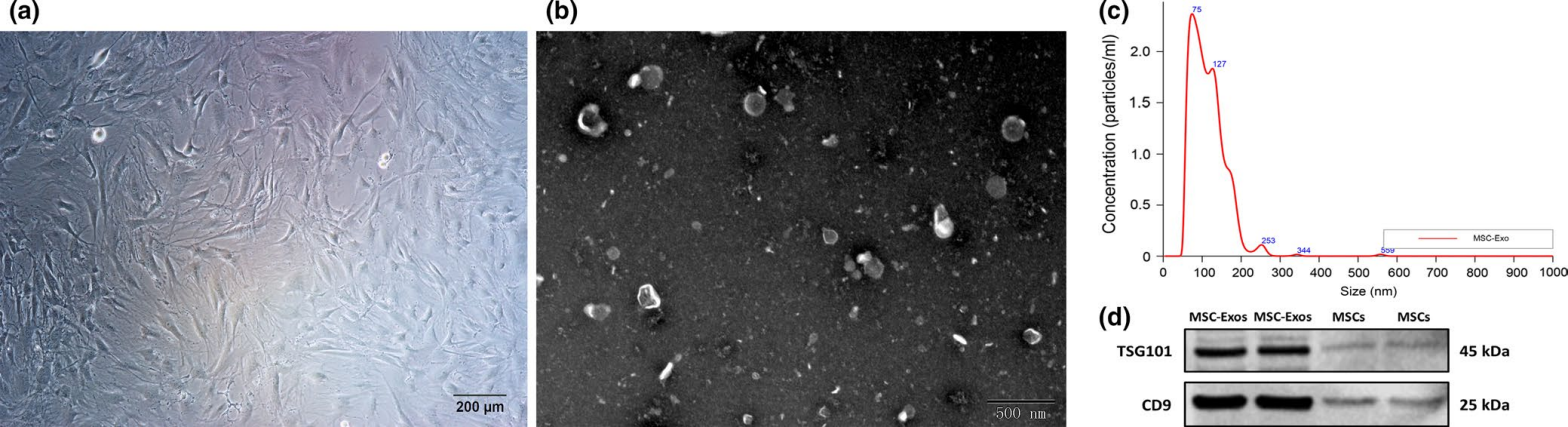
Characterization of mesenchymal stem cells (MSCs) and mesenchymal stem cell‐derived exosomes (MSC‐Exos). A, The morphology of MSCs, Scale bar = 200 µm. B, Transmission electron micrograph image of MSC‐ Exos, Scale bar = 500 nm. C, Particle size distribution of MSC‐Exos measured by Nanoparticle Tracking Analysis. D, Western blot results indicating positive expression for the TSG101, CD9 in the MSC‐ Exos

The morphology of MSCs was observed under a light microscope, which manifests fusiform, homogenous fibroblastic morphology (Figure 2A). Transmission electron microscopy showed that MSC‐Exos were cup‐shaped double‐layer membrane structure with a diameter in the range of 50‐200 nm (Figure 2B). Nanoparticle Tracking Analysis demonstrated that the average diameter of the particle was about 115 nm; the main peak of particle size was 74 nm; and the concentration of the particle was 1.11 × 10^10^ particles/mL (Figure 2C). WB analysis of MSC‐Exos showed the existence of exosome characteristic marker proteins TSG101 and CD9 (Figure 2D).

### 3.2 MSC‐Exos treatment improves erectile function

**Figure 3:**
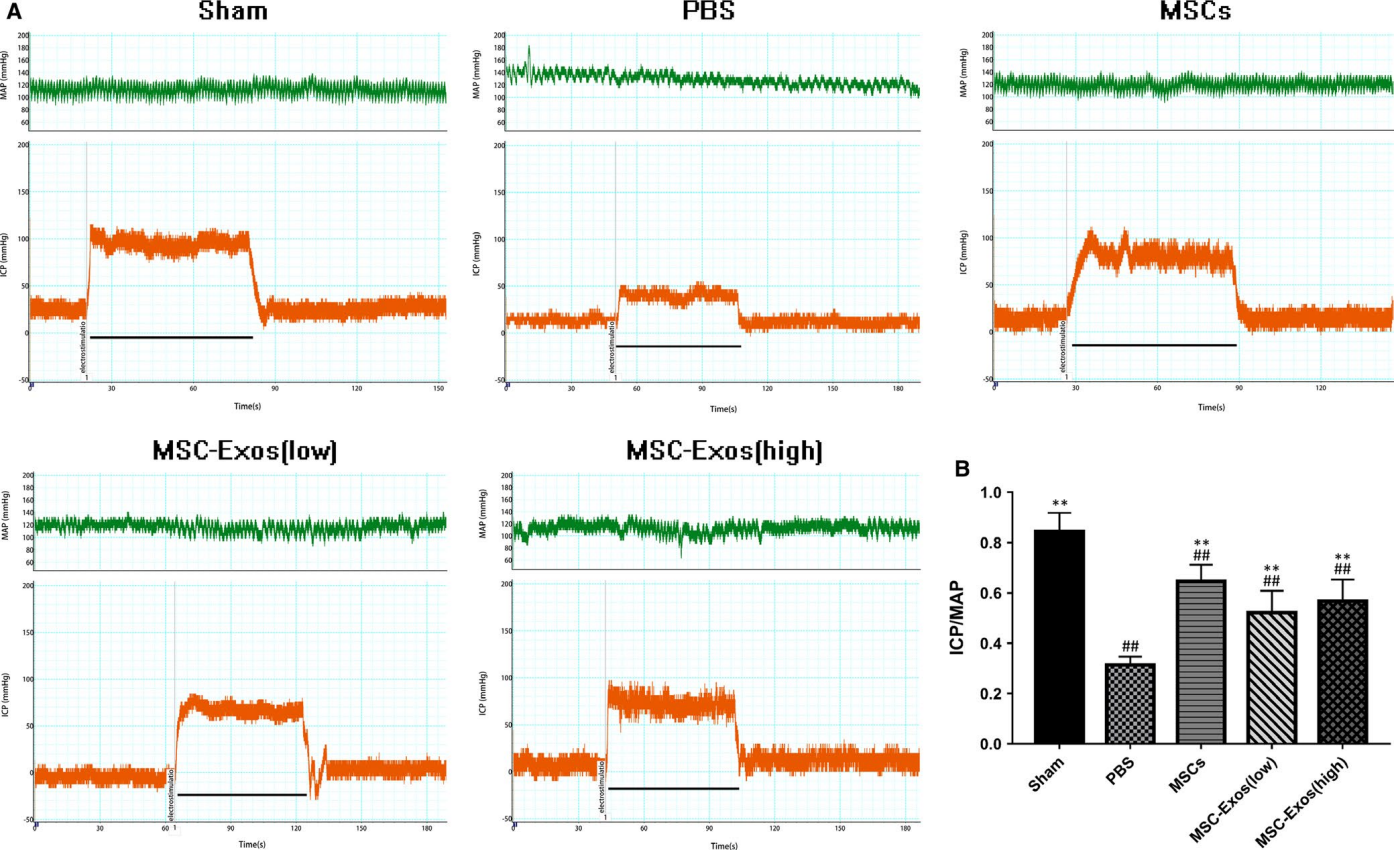
Intracavernous pressure (ICP) and mean arterial pressure (MAP) during cavernous nerve electrostimulation at the fourth week after intracavernous (IC) injection. A, Representative ICP responses for the sham group, PBS group, MSCs group, MSC‐Exos (low) group and MSC‐Exos (high) group. The marker represents the start time of electrostimulation; the bar denotes the 60 s cavernous nerve electrical stimulation. B, The ratio of maximal ICP to MAP is recorded. Each bar depicts the mean ± standard deviation from n = 6 animals per group. **P < .01 vs the PBS group, ##*P* < .01 vs the Sham group

Erectile function was assessed after 4 weeks of IC injection in all groups (Figure 3). The ratio of maximal ICP to MAP was 0.84 ± 0.07, 0.32 ± 0.03, 0.65 ± 0.06, 0.52 ± 0.08, 0.57 ± 0.08 in the sham group, PBS group, MSCs group, MSC‐Exos (low) group and MSC‐Exos (high) group, respectively. As expected, the sham group exhibited normal ICP waveforms and high ratio of ICP/MAP; the PBS group had the lowest values of ICP/MAP ratio, it is proved that bilateral internal iliac arterial ligation consistently resulted in ED. Compared with the PBS group; the ratio of treatment groups was significantly increased (*P* < .01 for all). These results indicated that MSC‐Exos could improve the erectile function of ED rats. Nevertheless, there was no statistically significant between MSC‐Exos (low) and MSC‐Exos (high) group (*P* = .74).

### 3.3 IC‐injected MSC‐Exos promote cavernous endogenous stem cells differentiate into cavernous sinus endothelial cells

**Figure 4:**
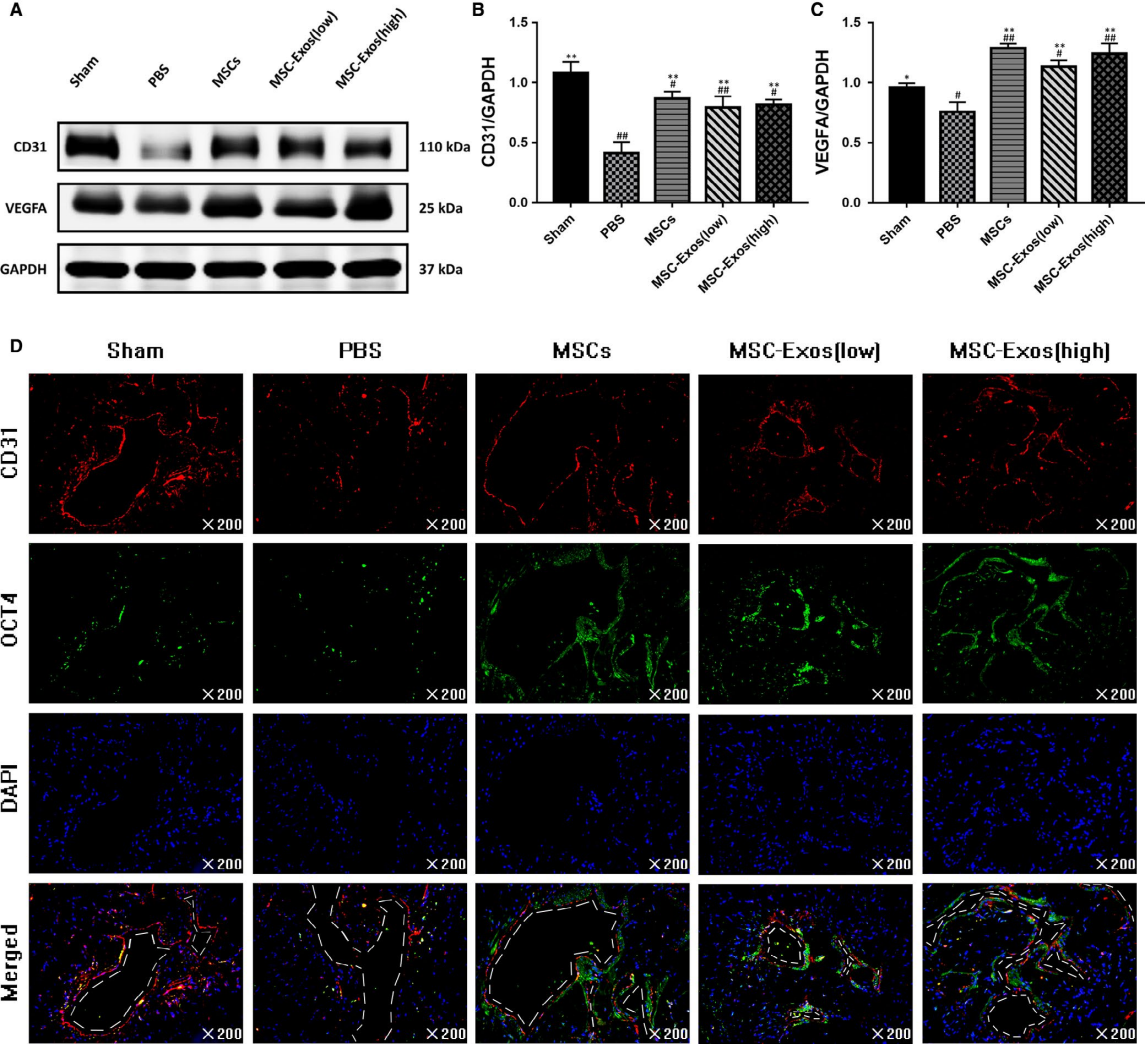
IC‐injected MSCs or MSC‐Exos promote cavernous endogenous stem cells differentiate into cavernous sinus endothelial cells. A, Representative images of western blots for CD31 and VEGFA in cavernosum from each group. B and C, Data are presented as the relative density of CD31 and VEGFA compared with that of GAPDH. Each bar depicts the means ± standard deviation from n = 6 animals per group. *P < .05 vs the PBS group, **P < .01 vs the PBS group, #*P* < .05 vs the Sham group, ##*P* < .01 vs the Sham group. D, Representative immunofluorescence staining of CD31 (red) and OCT4 (green) in a penile midshaft specimen from each group. The dotted line represents the contour of the cavernous sinus. Original magnification, ×200

As the WB result show (Figure 4A), the protein expression level of CD31 and VEGFA in the treatment groups was all significantly increased compared with those in the PBS group (*P* < .01 for all, Figure 4B,C). According to the histomorphometric analyses of IF (Figure 4D), we found that the positive expression of OCT4 increased in corpus cavernosum of the treatment groups, especially elevated OCT4 expression was observed around CD31 positive cavernous sinus endothelial cell, and this phenomenon is not obvious in the sham group and PBS group.

### 3.4 Effects of MSC‐Exos on NO/cGMP signalling pathway

**Figure 5:**
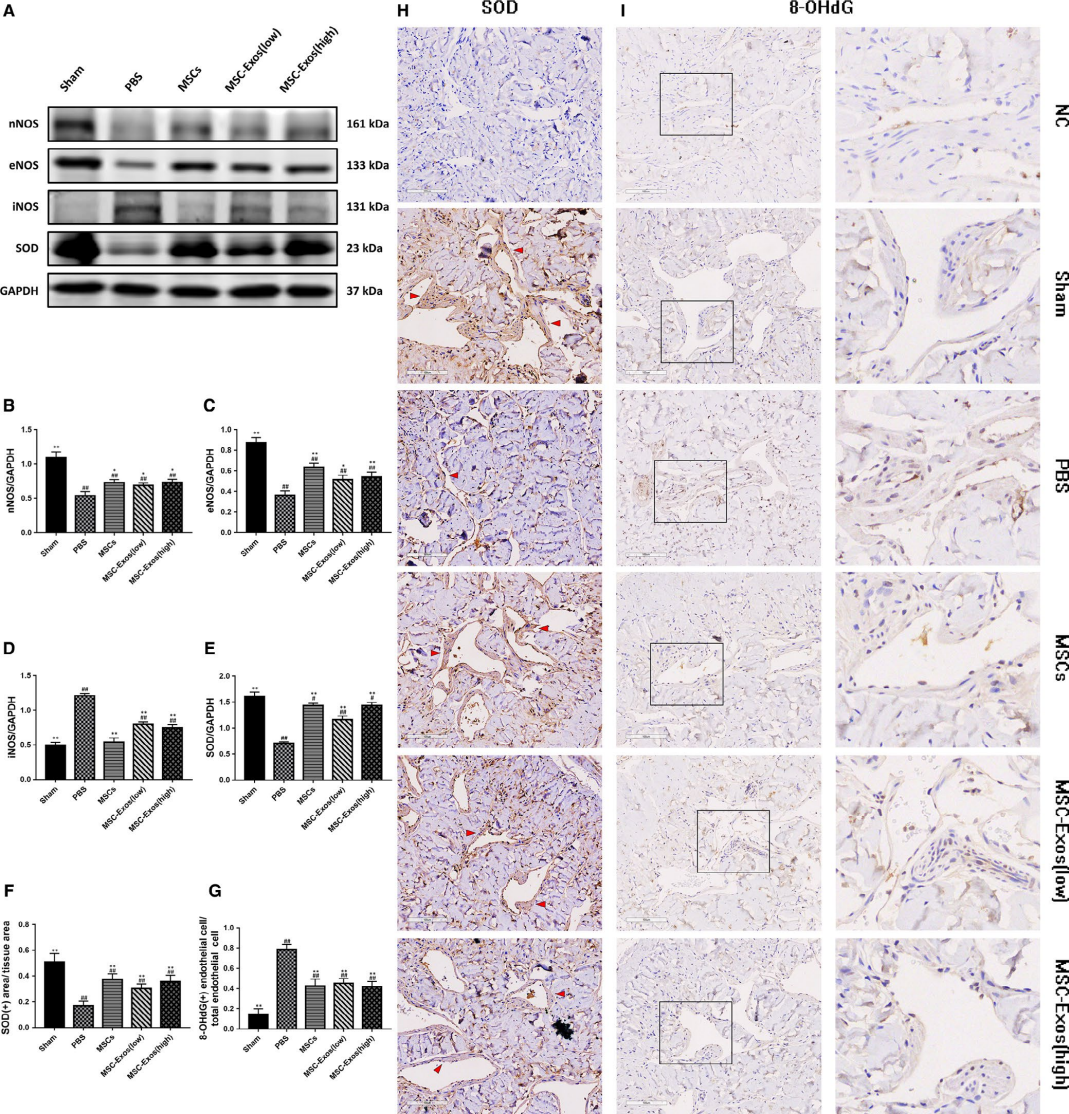
Transplantation of MSCs or MSC‐Exos influence the nitric oxide synthase (NOS) content in the corpus cavernosum and reduce the organization oxidative stress damage. A, Representative images of western blots for nNOS, eNOS, iNOS and SOD in cavernosum in each group. B‐E, Western blots' data are presented as the relative density of nNOS, eNOS, iNOS and SOD compared with that of GAPDH. F and G, Immunohistochemically semi‐quantitative data of the proportion of SOD positive expression area and 8‐OHdG positive expression endothelial cells. Each bar depicts the means ± standard deviation from n = 6 animals per group. *P < .05 vs the PBS group, **P < .01 vs vs the PBS group, #*P* < .05 vs the Sham group, ##*P* < .01 vs the Sham group. H and I, Immunohistochemical expression of SOD and 8‐OHdG, respectively, in corpus cavernosum. The negative control (NC) group used PBS to serve as the primary antibody. Red arrowheads refer to the area of positive tissue expression of SOD. Original magnification, ×200

In corpus cavernosum tissues, nNOS and eNOS are the dominant drivers of penile erection, which could catalyse the NO released during non‐cholinergic, non‐adrenergic neurotransmission and from the endothelium, thereby activating cyclic guanosine monophosphate (cGMP) signalling pathway.^22^ As the WB result show (Figure 5A), the levels of nNOS and eNOS in the treated groups were higher than those in the PBS group (*P* < .05 for all), but lower than the sham group (*P* < .01 for all; Figure 5B,C). The iNOS was also an important factor in erection. It was reported that the production of iNOS is closely related to the existence of inflammatory environment in the cavernosum.^23^ The data revealed that iNOS expression was higher in the PBS and MSC‐Exos groups than in the sham group (*P* < .01), but treat with MSC‐Exos could decrease the expression of iNOS in comparison with the PBS group (*P <* .01; Figure 5D).

### 3.5 Transplantation of MSC‐Exos reduce oxidative stress damage

Superoxide dismutase is an important antioxidant defence enzyme, which could catalyse the dismutation of the superoxide radical into ordinary molecular oxygen in living cells. Western blot and immunohistochemical staining for SOD was implemented to evaluate the enzyme content in corpus cavernosum tissue (Figure 5A,H). The SOD expression levels were considerably decreased in cavernosum tissue of the PBS group compared with the sham group (*P* < .01). Four weeks after treatment with MSC and MSC‐Exo, the expression of SOD was restored to varying degrees (*P* < .01 for all, Figure 5E,F). Beyond that, 8‐OHdG expression in cavernosum was evaluated for determining DNA oxidative stress damage (Figure 5I). The positive 8‐OHdG endothelial cells in ED rats were obviously increased whereas there was statistically significant decreased at the MSCs and MSC‐Exos groups when compared to the PBS group (*P* < .01 for all; Figure 5G).

### 3.6 MSC‐Exos treatment improves the ratio of smooth muscle to collagen and smooth muscle content in the corpus cavernosum

**Figure 6:**
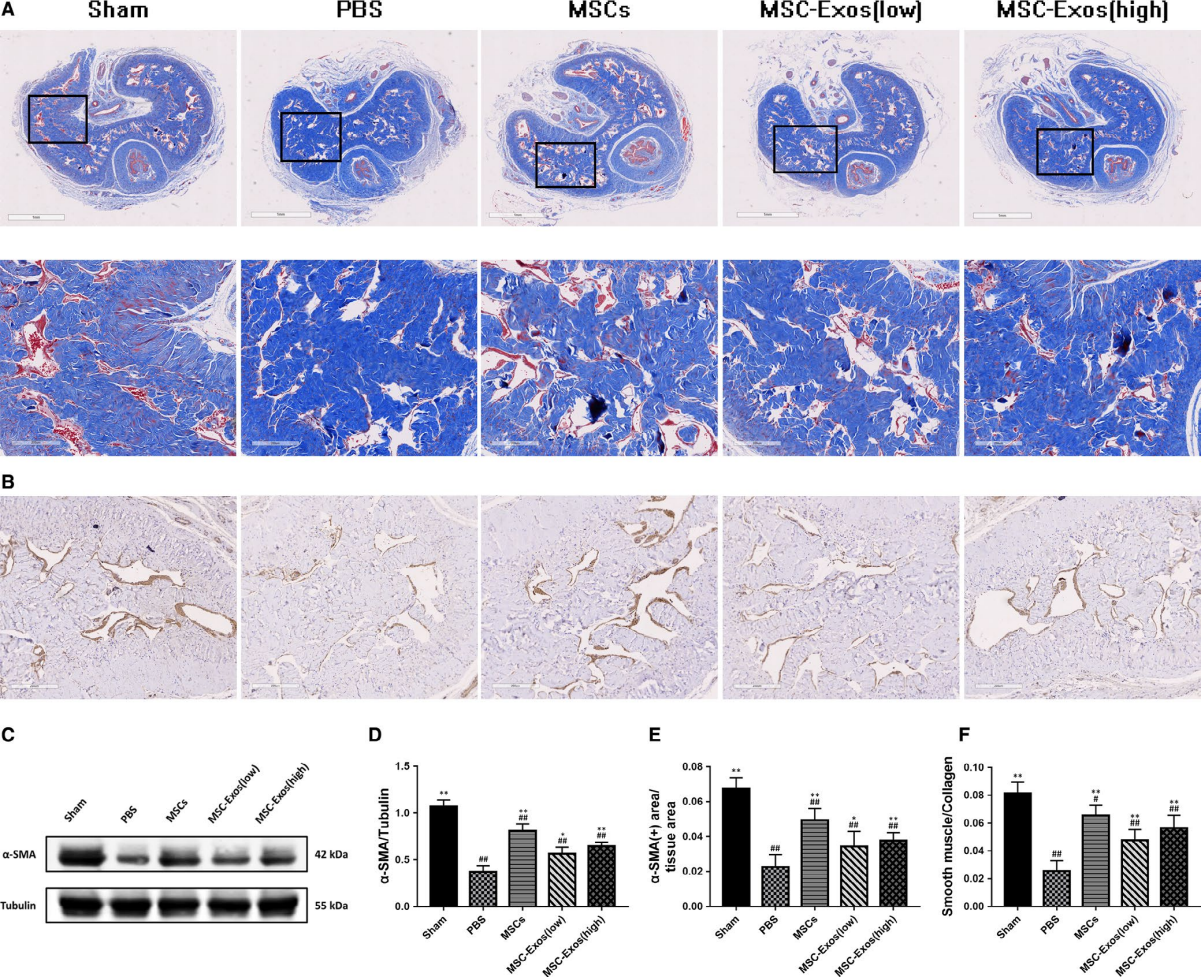
Treatment improves the ratio of smooth muscle to collagen and smooth muscle content in the corpus cavernosum. A, The smooth muscle (red) and collagen (blue) tissues stained by Masson's trichrome staining. Original magnification, ×20 and × 100. B, Immunohistochemical expression of α‐SMA in corpus cavernosum. Original magnification, ×100. C, Representative images of western blots for α‐SMA in cavernosum in each group. D, Western blots' data are presented as the relative density of α‐SMA compared with that of GAPDH. E, Immunohistochemically semi‐quantitative data of the proportion of α‐SMA positive expression area. F, The semi‐quantitative data of the ratio of smooth muscle area to collagen area. Each bar depicts the means ± standard deviation from n = 6 animals per group. *P < .05 vs the PBS group, **P < .01 vs the PBS group, #*P* < .05 vs the Sham group, ##*P* < .01 vs the Sham group

According to the statistical result of Masson's trichrome staining (Figure 6A), the ratio of cavernosum smooth muscle to collagen was 0.081 ± 0.008, 0.066 ± 0.007, 0.048 ± 0.008, 0.057 ± 0.009 in the sham group, MSCs group, MSC‐Exos (low) group and MSC‐Exos (high) group, respectively, which all higher than the PBS group (0.026 ± 0.07, *P* < .01 for all; Figure 6F). Corpus cavernosum smooth muscle content was detected by immunohistochemical staining and Western blot (Figure 6B,C). The penile tissue from the PBS group showed significantly lower α‐SMA‐positive staining area compared with the sham group (*P* < .01). Both MSCs and MSC‐Exos treat exhibited partial but significant restoration of smooth muscle content after bilateral internal iliac arterial ligation, and similar results were observed by WB analysis (Figure 6E,D).

### 3.7 The perimeter changes of cavernosum

**Figure 7:**
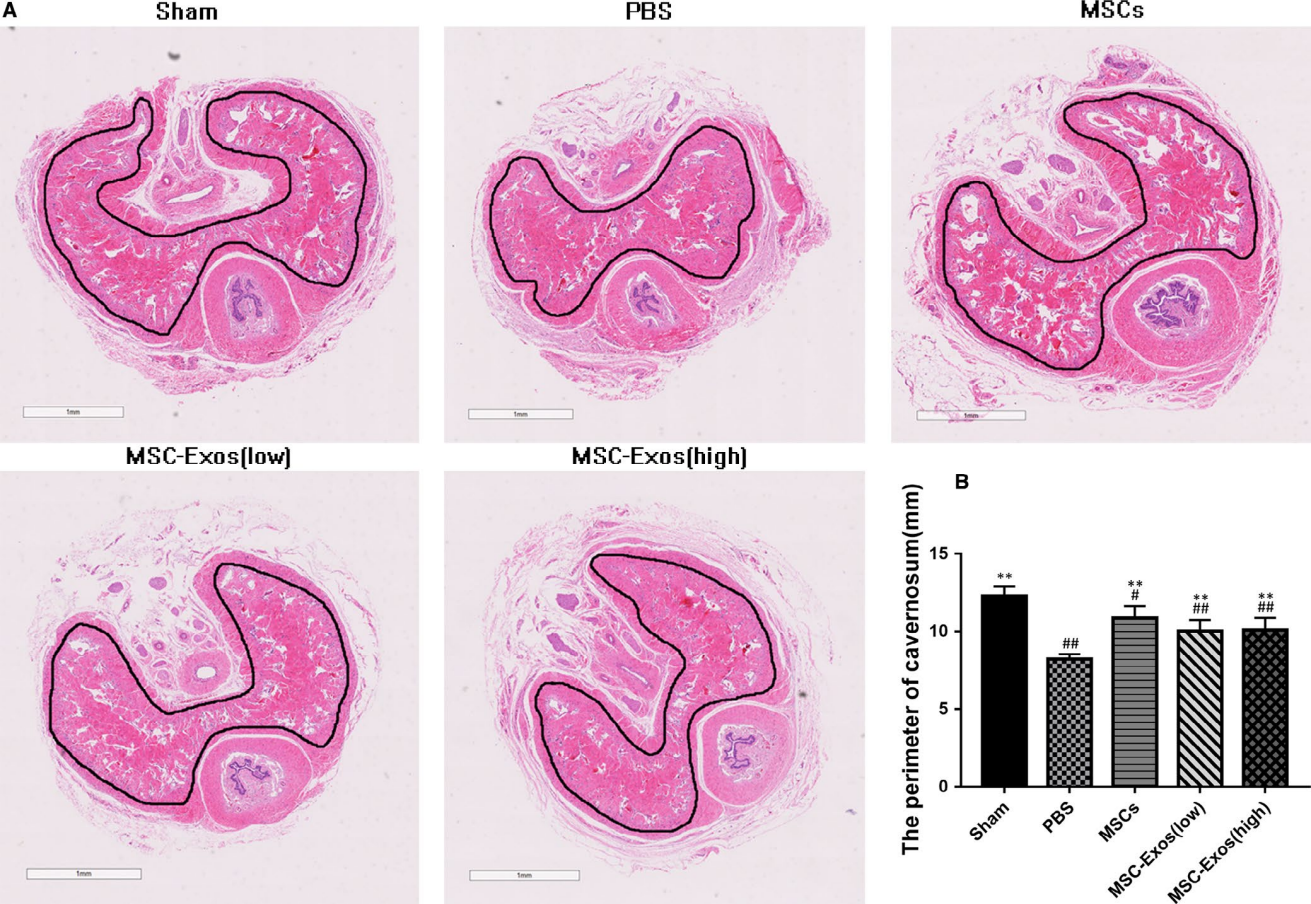
The perimeter changes of corpus cavernosum. A, Representative photomicrographs of haematoxylin and eosin (HE) staining in cavernosum which perimeter indicated with dark line. B, The perimeter of corpus cavernosum is recorded from each group. Each bar depicts the means ± standard deviation from n = 6 animals per group. **P < .01 vs the PBS group, #*P* < .05 vs the Sham group, ##*P* < .01 vs the Sham group

Bilateral internal iliac arterial ligation caused corpus cavernosum atrophy (Figure 7), compared with the sham group, the cavernosum perimeter of the PBS group was statistically decreased (*P* < .01). Treatment by MSC and MSC‐Exo could improve this pathological change at a certain extent after arterial ligation (*P* < .01 for all).

## 4 DISCUSSION

In the current study, we found that MSC‐Exos could effectively improve the erectile function in a rat model of arterial injury. More specifically, IC injection exosomes could ameliorate erection by promoting cavernous sinus endothelial formation and reduce the organization oxidative stress damage. Although MSCs are highly capable of differentiation, the paracrine mechanism of MSCs is currently considered to play a major role in therapy.^11^ Exosome of MSC is an important bioactive substance vector which contains multiple types of nucleic acids, lipids and proteins.^12^ They have been demonstrated to involved in multiple pathologic and physiologic processes, which include proliferation and differentiation,^24^ anti‐fibrosis,^25^ immunomodulation^26^ and tissue regeneration.^27^ In our study, exosomes were isolated from the culture supernatants of MSCs, and these pellets proved could ameliorate rat's erectile function independently with similar potency compared with the MSCs group.

Erectile dysfunction, a multifactorial condition with a complex neurovascular process, can be observed typically in patients with endocrinologic, neurogenic, vasculogenic, cavernosal diseases and drug‐induced.^28^ In order to simulate clinically common vascular ED, we established an arterial injury ED model by ligating the bilateral internal iliac artery of rats.^18^, ^29^ Despite the formation of vascular collateral circulation after ligation, the blood supply to the corpus cavernosum cannot be completely blocked, but the reduction of blood supply will cause the cavernosum tissue continues to be stimulated by the ischemic and hypoxic environment, thereby increasing the release of reactive oxygen species in the penis. Many studies have suggested that oxidative stress is the main factor of ED progress during penile ischemia,^6^, ^7^ which may lead to cell apoptosis, cavernosum vascular endothelium and nerve damage.^30^ It has been reported in the literature that exosomes released by stem cells can improve erectile function in a rat model of cavernous nerve injury and type 2 diabetes by anti‐apoptosis.^16^, ^17^

According to the histological analysis of penis 1 month after IC injection, we found that the possible mechanism of MSC‐Exo alleviating oxidative stress damage might be through the mobilization of cavernous endogenous stem cells to differentiate into endothelial cells. MSCs have been shown to promote tissue angiogenesis by paracrine the vascular endothelial growth factor (VEGF) or exosome containing VEGF and specific miRNA.^31^, ^32^ Vascular endothelial growth factor is a highly specific factor that has been shown to stimulate endothelial cells migrations and differentiation. It is reported that VEGF alone or combined with other factors can induce stem cells to undergo to endothelial cell differentiation. These cytokines have fully proved to have the ability to induce stem cell differentiation in vitro. 33 As a marker of stem cell, the Octamer‐binding transcription factor 4 (OCT4) not only expressed in embryonic stem cells, but also reported to widely exist in adult stem cells in recent years. It is related to the pluripotency maintaining and regulating the differentiation ability of stem cells.^34^, ^35^ Nolazco G et al reported that the quiescent endogenous stem cell was detected in the corpora cavernosa and proved to have the ability of differentiation after induction in vitro.^36^, ^37^ In our study, we found that the expression of VEGF increased in the treatment group meanwhile the number of OCT4‐positive cells around vascular endothelium increased, suggesting that newly formed vascular endothelial cells might be derived from cavernous endogenous stem cell differentiation. Similar studies have been reported in stem cell therapy for myocardial injury.^38^ Based on the above researches, we speculate that endogenous stem cells in the corpus cavernosum can be recruited and mobilized to participate in tissue repair during injury, and this process is enhanced after MSC‐Exo treatment. Nevertheless, since for matured cells that have been differentiated, OCT4 is not expressed basically,^34^ while the expression of CD31 prove that endothelial cells have differentiated.^39^ Therefore, in the newly differentiated endothelial cells, the co‐localization of CD31 and OCT4 positive expression cannot be achieved, the differentiation process of stem cells is difficult to track in vivo. This hypothesis still needs to be verified by inducing differentiation of stem cells extracted from corpus cavernosum in vitro.

Although promoting angiogenesis is not the only way for MSC‐Exo therapy to alleviate oxidative stress damage,^40^, ^41^ it is undeniable that the repair of cavernous sinus endothelial cells after injury can be regarded as an important factor that cannot be ignored in improving erectile function. First of all, the release of NO which activates cGMP signalling pathway needs to catalysed by NOS. Cavernous sinus endothelial formation and local blood supply improved could increase the level of eNOS and nNOS in the cavernosum, conversely, high levels of iNOS in the untreated group indicates the presence of inflammatory reaction.^42^ In addition, we observed corpus cavernosum smooth muscle content was increased after treatment. On the one hand, adequate blood supply can provide sufficient nutrition for cavernosum smooth muscle cells. On the other hand, one of the advantages of MSC‐Exos in treatment of ED may be their ability to anti‐apoptotic of cavernosum smooth muscle cells.^16^

Penile erection is a comprehensive result of nerve mobilization, arterial blood supply and cavernous blood storage, which requires the structural integrity and functional coordination of the cavernous sinus, cavernous smooth muscle and cavernous nerve. Although compared with Ouyang et al,^16^, ^17^ a similar phenomenon was observed in our research, which MSC‐Exos treatment could improve the smooth muscle content in the corpus cavernosum. However, Ouyang's research confirmed that exosomes improve erection by directly acting on cavernous smooth muscle cells, while our study demonstrates that exosomes alleviate erectile dysfunction mainly by promoting the formation of cavernous sinus vascular endothelium, which may bring a series of positive effects such as reducing tissue oxidative stress damage. From another perspective, we supplement and confirm that exosomes derived by stem cell not only act on a particular type of cells, but also can improve the organ function by affecting the internal environment of the corpus cavernosum.

At present, the main methods for clinical treatment of arterial ED include oral PDE5is, cavernous injection, penile vascular reconstruction, Balloon Angioplasty, and penis prosthesis implantation. However, oral PDE5is are difficult to achieve satisfactory results for the ED patients with severe organic diseases. Because the surgery is invasive, and it is poorer prognosis when the lesion is located in a small vessel such as the internal pudendal artery, thus it is generally not the preferred treatment. Compared with these treatments and the stem cell therapy, IC injection exosome therapy has many advantages, mainly include the following: (a) exosome is more stable than cell, easy to store and manage. (b) immune rejection is not easy to occur, and the risk of tumour formation is low compared with the stem cell therapy. (c) it has strong plasticity and is easy to be absorbed by receptor cells. (d) complications such as bleeding and nerve injury less than open surgery. Nevertheless, it is undeniable that the current research still has certain limitations. First, although we have setted up different treatment concentration gradients of MSC‐Exo, the therapeutic effect has not been found significant improvement. It may be a result of the limited uptake capacity of receptor cells. We assume that multiple dosing can improve the therapeutic effect and need to be further validated. Second, we found that the effect of exosome treatment on improving erection was related to the increase of VEGF content in tissues; it may because stem cells can promote tissue angiogenesis by secreting VEGF‐rich exosomes.^43^, ^44^ However, due to the inclusions of exosome are very complex, there are also related researches that attribute its active ingredients to miRNAs. As a key factor in exosomes facilitating VEGF signalling, miRNA‐126 in MSC‐Exo has been proved to have a great effect on promoting angiogenesis.^45^, ^46^ All of this suggests that different inclusions in exosomes may synergistically enhance the therapeutic effect, the specific mechanisms of the MSC‐Exo involved in the repair of injury remain need to be further identified.

## 5 CONCLUSIONS

Our results provide the evidence that intracavernous injection MSC‐derived exosomes could ameliorate erectile function by reducing the oxidative stress damage of corpus cavernosum in an arterial injury‐induced ED rat model, which further has great potential in the clinical treatment of severe arterial injury ED.

## CONFLICT OF INTEREST

The authors confirm that there are no conflicts of interest.

## AUTHOR CONTRIBUTIONS

Yangzhou Liu: experimental design, manuscript writing and data analysis; Shankun Zhao: experiment modelling, manuscript writing and data analysis; Lianmin Luo: experiment modelling and data collection; Jiamin Wang and Zhiguo Zhu: histology examination and data collection; Qian Xiang and Yihan Deng: data analysis; Zhigang Zhao: experimental design and manuscript editing.

## Data Availability

The data that support the findings of this study are available from the corresponding author upon reasonable request.
